# Prevalence and Correlates of Likely Major Depressive Disorder among the Adult Population in Ghana during the COVID-19 Pandemic

**DOI:** 10.3390/ijerph18137106

**Published:** 2021-07-02

**Authors:** Medard Kofi Adu, Lauren J. Wallace, Kwabena F. Lartey, Joshua Arthur, Kenneth Fosu Oteng, Samuel Dwomoh, Ruth Owusu-Antwi, Rita Larsen-Reindorf, Vincent I. O. Agyapong

**Affiliations:** 1 Department of Psychiatry, Faculty of Medicine and Dentistry, University of Alberta, Edmonton, AB T6G 2R3, Canada; medard@ualberta.ca (M.K.A.); agyapong@ualberta.ca (V.I.O.A.); 2 Dodowa Health Research Centre, Research and Development Division, Ghana Health Service, Dodowa P.O. DD1, Ghana; lauren.jean.wallace@gmail.com; 3 Chan School of Public Health, Harvard University, Boston, MA 02115, USA; fosulartey@yahoo.com; 4 Public Health Unit, Komfo Anokye Teaching Hospital, Kumasi P.O. Box 1934, Ghana; arthureuk@yahoo.co.uk; 5 Ashanti Regional Directorate, Ghana Health Service, Ashanti Region, Kumasi P.O. Box 1934, Ghana; otengkenneth24@gmail.com (K.F.O.); dwomohsamuel6060@gmail.com (S.D.); 6 Department of Psychiatry, Komfo Anokye Teaching Hospital, Kumasi P.O. Box 1934, Ghana; rucha_2001@yahoo.com

**Keywords:** major depressive disorder, depression, mental health, COVID-19, pandemic, Ghana

## Abstract

Background: Emerging research suggests that the novel coronavirus disease (COVID-19) pandemic and associated public health restrictions have caused psychological distress in many contexts. In order for public health authorities and policy makers to effectively address the psychological distress associated with the pandemic, it is important to determine the prevalence and correlates of mental disorders, including depression. Objectives: We aimed to determine the prevalence, and demographic, social, clinical and other COVID-19 related correlates of major depressive disorder symptoms among the general population in Ghana during the COVID-19 pandemic. Method: The study was a cross-sectional survey using online data collection methods. The survey assessed demographic, social and clinical variables as well as COVID-19 related variables. Major depressive disorder symptoms were assessed using the Patient Health Questionnaire-9. The survey link was distributed primarily through WhatsApp-based platforms. Data were analyzed using descriptive and inferential statistics. Results: The overall prevalence of likely MDD symptoms among the sample population was 12.3%. Variables such as employment, loss of jobs during the pandemic and rate of exposure to COVID-related news were independently and significantly associated with the likelihood that respondents had likely MDD. Variables such as gender, relationship, housing status and having a family member or friend who was sick from COVID-19 were not independently significantly associated with the likelihood that respondents had likely MDD, when all other factors in the model were controlled. Conclusion: This study has identified the prevalence and correlates of depression symptoms in Ghana during the COVID-19 pandemic. There is the urgent need for mental health policy makers and the government of Ghana to have policies in place to alleviate the potential threat to the mental health of the population.

## 1. Introduction

The novel coronavirus disease (COVID-19) was initially identified in December 2019 in Wuhan, the capital of Hubei Province, China [1]. As of 22 May 2021, there had been over 166.3 million confirmed cases of COVID-19 globally, including over 3.4 million deaths. In Ghana, as of the same date, there had been 93,583 confirmed cases of COVID-19 and 783 deaths [2]. The COVID-19 pandemic, like any other event that causes emotional, physical and psychological distress, can be considered a traumatic event [3]. The pandemic has created new stressors and disruptions to daily living for people around the world. This includes concern for one’s own health and well-being and that of one’s close relations, and constant exposure to information about the pandemic and its adverse effects. In addition, the policy measures implemented by authorities to limit the spread of the disease, including stay at home orders, have resulted in the loss of control and personal freedoms, decreased social contact, conflicting messages from authorities, sudden changes in plans for the immediate future and adverse economic effects. The lives of individuals infected with COVID-19 may also be adversely affected by quarantine and isolation measures, and the experience of stigma [4] from family members and communities [5]. Traumatic events and the associated socioeconomic problems they present have a direct correlation with mental health problems and are thought to increase vulnerability to and prevalence of conditions such as depression [6,7].

Recent studies in several contexts have reported an upsurge of depression in the general population during the COVID-19 pandemic, although the prevalence of depression varies widely across contexts. In Turkey, the prevalence of depression during the COVID-19 pandemic was reported as 23.6% [8], while it was 25.1% among the general population during the lockdown [9]. In a cross-sectional survey of the general public who were subscribers of the Text4Hope program in Alberta, Canada during the COVID-19 pandemic, the prevalence of depression among the population was 44.0% [10]. In contrast, the prevalence of depression was 82.4% among university students during the COVID-19 pandemic in Bangladesh [11]. 

Studies from previous epidemics and early findings from the COVID-19 pandemic have highlighted the psychological and mental health impact of health emergencies on healthcare workers across the globe [12]. According to a recent systematic review, the estimated prevalence of depression among healthcare workers globally during the COVID-19 pandemic is 25% [13]. Among health practitioners in China, where the infection started, the prevalence of depression ranged from 3.7% to 48.3% [5,14,15,16,17,18] and 6.46% to 50.7% [19,20]. Another study among healthcare workers in Kenya reported that 53.6% and 9.2% of healthcare workers exhibited mild and severe depressive symptoms respectively [21]. 

High rates of depression among healthcare workers may be a result of inadequate personal protective equipment and training on infection prevention and control measures [22], in addition to the fear of getting infected and limited social support [23]. Other variables such as the public health measures put in place to control disease spread may be responsible for the high prevalence of depression in the general population during health emergencies. According to another study, the higher rate of depression during the SARS outbreak in Canada was partly attributed to the duration and uncertainty of the lockdown during that era [24]. The lockdown measures put in place across the globe, including in Africa, could therefore to some extent contribute to a higher prevalence of depression during the COVID-19 pandemic [25]. 

Suspected COVID-19 symptoms [26], high mortality rates [14], lower immunity [27], being close to an infected person [28], having an underlying medical condition [29], limited access to routine medications for those with existing mental health problems and limited support for those affected with domestic violence [30,31] have also been associated with depression. Some non-health related variables such as younger age and being female [14] have also been found to be associated with a higher prevalence of depressive symptoms during the COVID-19 pandemic [15]. 

Other socioeconomic issues such as uncertainty about the job market, career progression and higher levels of media exposure [32] may also account for a higher prevalence of mental conditions such as depression. The nature of social support during a health emergency can also impact mental health. For example Sub-Saharan African countries such as Ghana are naturally designed with a firm sense of community in which people depend on one other for social support [27]. Lockdown measures may lead to a breakdown in available community supports. 

Depression may have an adverse impact on the immune system response and can also worsen the prognosis of COVID-19 in affected individuals [33] by reducing adherence to treatment [34]. Without prompt interventions, depressive symptoms may evolve into long term depression [35,36]. Studies indicate that patients could display persistent depression even years after an outbreak of infectious disease such as COVID-19. Among patients with Severe Acute Respiratory Syndrome (SARS), the prevalence rate of depression was 18% within 4 weeks after discharge [37] and 15.6% after 30 months of the SARS pandemic [38]. Prolonged depression may result in severe cognitive and social dysfunction [39] and suicidal ideation. However, the surge in depressive symptoms during a pandemic such as COVID-19 can be reduced by the provision of timely psychological evaluations and prompt mental health services [20]. 

In order for public health authorities and policy makers to effectively curb the prevalence of depression and to enhance the recovery of COVID-19 patients, it is essential to understand the pattern of depression and its characteristic features in diverse contexts [34]. The goal of this study is to determine the prevalence and the sociodemographic, clinical and other COVID-19 related correlates for likely Major depressive disorder symptoms amongst the general population in Ghana during the COVID-19 pandemic. To our knowledge, this is the first study in Ghana to evaluate the prevalence and correlates of clinically meaningful depression in the general population. The current study contributes to the growing body of evidence on the psychological impact of the COVID-19 pandemic in Sub-Saharan Africa.

## 2. Study Site

Ghana’s first case of COVID-19 was confirmed on 12 March 2020. The government of Ghana announced social distancing measures and travel restrictions on 10 March, including the suspension of international travel by public officials, restrictions on public gatherings and the closure of schools. Starting on 22 March 2020 all air, land and sea borders were closed, and beginning on 27 March, a three-week partial lockdown was imposed in parts of Greater Accra and Ashanti Regions: densely populated areas which have been the epicenters of the epidemic. Ghana’s first wave of COVID-19 spanned from approximately 12 March 2020 to 30 September 2020. Following a decline in case numbers, a phased approach to easing the initial restrictions began on 5 June 2020, including lifting of some restrictions on social gatherings and religious services, and the decision was taken by the government to re-open schools. The second wave of the epidemic in Ghana began in January 2021, following the election period and Christmas festivities. 

## 3. Methods

The study was a cross-sectional survey primarily aimed at collecting data on likely major depressive disorder and its relation to the COVID-19 pandemic. The survey included demographic, social, clinical and COVID-19-related variables and was adapted from survey questions used in Canada to gather baseline data from Text4Hope (a supportive text message program) subscribers during the COVID-19 pandemic [10,17,40,41,42,43,44]. Major depressive disorder symptoms were assessed using the Patient Health Questionnaire-9 (PHQ-9) [45]. The PHQ-9 is a 9-item validated instrument (associated with a Cronbach’s alpha of 0.89) which is used to diagnose and measure the severity of depression in general medical and mental health settings. Each of the 9 questionnaire items is scored between 0 (not at all) to 3 (nearly every day). Higher scores on the scale indicate higher levels of depression (for likely major depressive disorder or MDD; PHQ-9 ≥ 10) [45].

The survey was hosted on the Qualtrics XM platform as an online questionnaire. The survey link was distributed primarily through WhatsApp-based platforms. Several WhatsApp groups were identified and some of these groups included nurses’ groups, doctors’ groups, student groups of high schools and the general public. Despite the anonymous nature of the survey, respondents were asked to provide their contact information if they would like to receive mental health counselling. The local mental health team at the Komfo Anokye Teaching Hospital contacted respondents who provided their contact information to do a mental health assessment and to offer support and connections to local mental health services. Data were collected from 8 November 2020 to 2 January 2021. With an estimated population in Ghana of 30 million people, using the sample size calculator (available online: https://www.surveymonkey.com/mp/sample-size-calculator/ accessed on 1 May 2021), the sample size needed to estimate the prevalence for likely MDD with a 95% confidence interval and a 3% margin of error was 1068. 

Ethical approval was obtained from the Ghana Health Survey Ethics Review Committee [*GHS-ERC 027/08/20*]. Upon opening the survey, respondents were provided with a specific question explaining the purpose of the survey and seeking their consent to which if they responded ‘No’, the survey was immediately terminated. There was a footnote throughout all the sections of the survey which prompted respondents to simply exit the survey if at any time they were not comfortable proceeding. Data for all respondents who did not complete the survey by clicking submit were destroyed.

## 4. Data Analysis 

Data were analyzed using the Statistical Package for Social Sciences (SPSS) version 26. Descriptive statistics for demographic characteristics are reported in numbers and percentages. Cross-tabular univariate analyses with Chi-square or Fisher’s exact tests were used to explore the relationship between the categorical variables in the study and moderate/high depression symptoms. Variables with a statistically significant relationship (*p* < 0.05, two-tailed) and variables that trended toward significance (0.05 ≤ *p* ≤ 0.10, two-tailed) with the likelihood of moderate/high depression symptoms on a univariate analysis were the only variables analyzed using logistic regression modelling. Preliminary checks were conducted to confirm all the assumptions of the logistic regression, including the presence of an adequate sample size, absence of multicollinearity and absence of outliers. Thus, before performing the logistic regression analysis, correlation diagnostics were performed to ensure that high inter-correlations among the predictor variables were avoided. The Spearman’s correlation coefficients were less than 0.7 for all included variables which suggested no multicollinearity existed among the independent variables. 

Odds ratios from the binary logistic regression analysis were examined to determine the association between each of the variables in the model and the likelihood of individuals reporting moderate/high depression symptoms, controlling for the other variables in the model. For each of the variables, the first variable option was used as the reference against which other variable options were compared. 

## 5. Results

Table 1 indicates that respondents were fairly balanced between males and females, with the majority residing in the Ashanti region of Ghana, having a university or college degree, being married and employed in a government agency. About one in five respondents reported they received absolute support from an employer, the government of Ghana, spiritual/traditional leaders and family and friends and the proportions of respondents were fairly balanced among the different age categories. Overall, just one in twenty respondents indicated they had sought counselling and one in ten indicated they had received counseling during the pandemic. The proportions of respondents were also balanced among the different age categories. 

Table 2 suggests that about one in twenty respondents lost their jobs during the pandemic, and the proportions were balanced between the different age ranges. Overall, about two thirds of respondents watched television images and listened to radio stories related to sickness and death from COVID-19 with the smallest proportion of respondents in these categories being 25 years and younger. Seven out of ten respondents had been fearful of contracting the COVID infection, with the highest proportion aged 41–60 years. Three out of ten respondents had a family member or close friend contract the COVID-19 infection, and two out of ten respondents had had to isolate or quarantine due to symptoms, recent travel or contact with someone who may have COVID-19. Those aged 26–60 had a higher proportion of respondents belonging to the latter two of these categories compared to respondents in the other two age groups.

Table 2 suggests that overall, the prevalence of likely MDD in our sample was 12.3%. No respondent aged over 60 years old met the criteria for likely MDD, and the prevalence for likely MDD was highest among those aged 26–40 years.

## 6. Univariate Analysis

The association between all sociodemographic and COVID-19 news exposure and related variables and the likelihood that respondents had likely MDD is illustrated in Table 3 and Table 4. The results in Table 3 and Table 4 suggests that gender, employment and housing status were significantly associated (*p* ≤ 0.05) with the likelihood of likely MDD. For example, respondents who were female, unemployed, lived with family or friends and those who did not work in the health sector had a higher prevalence of likely MDD compared to respondents with other characteristics within the same demographic variables. Other variables such as being fearful of contracting COVID-19, frequency of exposure to COVID-19 related news on the internet or social media, radio and TV and loss of job during the pandemic were significantly associated with likely MDD.

## 7. Logistic Regression 

The results of the logistic regression predicting likelihood for respondents to present with likely MDD are as presented in Table 5. The full model containing all twelve predictors was significant, *Χ*^2^ (32, *N =* 485) = 89.75, *p* < 0.001, indicating the model was able to distinguish between individuals who reported moderate/high depression symptoms versus those who reported low depression symptoms. The model explained between 16.6% (Cox and Snell R^2^) and 32.1% (Nagelkerke R^2^) of the variance. Additionally, 90.1% of all cases were correctly classified. As shown in Table 5, respondents who were self-employed were 0.15 times less likely to have likely MDD compared to respondents who were employed by a government agency. In other words, respondents who were employed by a government agency were 6.67 (1/0.15) times more likely to present with likely MDD compared to those who were self-employed. Similarly, respondents who listened to COVID-19 related news on the radio every other day and once a week were respectively 0.37 and 0.11 times less likely to present with likely MDD compared to respondents who listened daily to COVID-19 related news items on the radio. This implies that respondents who listened daily to COVID-19 related news items on the radio were 2.7 (1/0.37) and 9.1 (1/0.11) times more likely to present with MDD symptoms compared to respondents who listened to COVID-19 related news every other day and once a week, respectively. Conversely, Table 5 suggests that respondents who watched images about COVID-19 related sicknesses and deaths on TV at most once a week were 3.1–3.8 times more likely to present with likely MDD symptoms compared to respondents who watched such images on TV daily. Respondents who lost their job during the COVID pandemic and those who did not have a job were 0.03 times less likely to present with likely MDD compared to respondents who did not lose their jobs during the pandemic. This means that respondents who lost their jobs were 30 (1/0.03) times more likely to present with MDD symptoms compared to respondents who did not lose their jobs and those who did not have jobs before the pandemic. Finally, respondents who were not interested in receiving counselling during the pandemic were 0.39 times less likely to present with likely MDD compared to respondents who expressed they would like to receive counselling. In other words, respondents who were interested in receiving counselling were 2.56 (1/0.39) times more likely to present with likely MDD compared with respondents who were not interested in receiving counselling. Gender, relationship and housing status, working in healthcare, having a family member or friend who is sick from COVID-19, fear of contracting a COVID-19 infection and frequency of reading internet and social media posts related to COVID-19 were not independently significantly associated with likely MDD, when all other factors in the model were controlled. 

## 8. Discussion

The purpose of this survey was to determine the prevalence of major depressive disorder symptoms and their relationship to sociodemographic, clinical and other COVID-19 related correlates among the general population in Ghana during the COVID-19 pandemic. The overall prevalence of likely MDD symptoms among the sample population was 12.3%. Respondents who were employed by a government agency were 6.67 times more likely to present with symptoms of MDD compared to those who were self-employed. Those who lost their jobs during the pandemic were 30 times more likely to present with MDD compared to those who did not lose their jobs. Similarly, the rate of exposure to COVID-19 related news also influenced participants’ chances of presenting with symptoms of MDD; those who listened daily to COVID-19 news on the radio were 2.7 and 9.1 times more likely to present with symptoms of MDD compared to others who did so every other day and once a week, respectively. Variables such as gender, relationship status, housing, working in healthcare and having a friend or family member who was sick with COVID-19 were not independently significantly associated with the likelihood that a respondent had likely MDD. 

Empirical evidence on national prevalence and correlates of mental disorders like depression in low and middle-income countries, particularly in Africa, is limited and the situation in Ghana is no different [46]. Although many studies on depression have been undertaken in Ghana, most have focused on specialized populations. For instance, studies of infertile women, geriatric depression and university studies report prevalence rates of 62%, 37.8% and 39.2%, respectively [47,48,49]. The severity, chronic and stress-provoking nature of the conditions in these specialized populations may account for their comparatively high prevalence rates of depression compared to the lower rate observed in our study.

The 12.3% prevalence of likely MDD symptoms among the general population in Ghana is higher than the 6.5% prevalence rate in China reported by Wang et al. (2020) at the initial stages of the COVID-19 pandemic, and the 9.2% prevalence rate reported in the general population of Cyprus about two weeks into the implementation of restrictive government measures [50]. However, the prevalence of likely MDD symptoms we observed in Ghana is lower than that reported in Canada and the USA (14.8–44.0%) in those settings [8,9,16,41]. Factors that could potentially contribute to the differences in the prevalence rates for likely MDD in each of these jurisdictions compared to Ghana include: differences in the restrictive measures put in place by governments, rates of infection, hospitalizations and deaths from COVID-19, economic and financial impact of the pandemic and the social protections available to citizens. Participants who were employed by a government agency were 6.67 times more likely to present with likely MDD compared to those who were self-employed. In the Ghanaian context, respondents who are employed by a government agency had financial security during the pandemic compared to those who were self-employed. For example, teachers continued to draw salaries even though schools were closed for a prolonged period during the early phase of the pandemic, including during the data collection period. In contrast, operators of private educational institutions and small businesses were offered no financial support from the state. Our results are therefore in contrast with previous studies that indicate that financial difficulty has a negative impact on the well-being of the self-employed especially in this COVID-19 period [51,52]. Notwithstanding the fact that self-employed people are more likely to experience major financial concerns, evidence in literature has it that they almost always report being satisfied with their well-being [53], which could account for our results. 

Our study also suggests that respondents who lost their jobs were 30 times more likely to present with MDD symptoms compared to respondents who did not lose their jobs and those who did not have jobs before the pandemic. Some of these job losses may have been a direct result of public health measures such as the lockdown put in place by authorities or other larger economic impacts of COVID-19 on the national and global economy. Similarly, some previous studies report that involuntary unemployment yielded a higher prevalence of depressive symptoms due to the anguish about the loss of job and uncertainty of future job stability [54,55].

Region of habitation was not significantly associated with the likelihood of presenting with MDD symptoms. However, our results suggest that the prevalence of likely MDD was higher in the two most densely populated regions of Ghana, the Greater Accra and Ashanti regions (17.1% and 11.6%, respectively) compared to those who lived in the other 14 regions combined (6.9%). The more densely populated the region, the higher the prevalence was for likely MDD. Although not statistically significant, this trend is consistent with the results of a study in Bangladesh which found an elevated prevalence of depression in the capital city and the populated districts near the capital during the COVID-19 pandemic [56]. Densely populated regions are more likely to have higher rates of COVID-19 infection. In addition, it may be more difficult to adhere to preventive public health measures in these settings. Both of these issues could lead to higher levels of stress in urban populations and increase the likelihood of individuals developing serious mental health problems like depression. This notwithstanding, region of residence did not show an independent association with likely MDD symptoms when all other factors were controlled for in this study. 

Respondents who listened daily to COVID-19 related news items on the radio were 2.7 and 9.1 times more likely to present with MDD symptoms compared to respondents who listened to COVID-19 related news every other day and once a week, respectively. This finding is consistent with other studies that identified high exposure to news on the radio and other social media platforms relating to COVID-19 as the major cause of stress and depressive symptoms [15,57]. In contrast, this study also suggests that respondents who watched images about COVID-19 related sicknesses and deaths on TV at most once a week were 3.1–3.8 times more likely to present with likely MDD symptoms compared to respondents who watched such images on TV daily. These conflicting findings suggest that further research is needed to confirm the correlation between COVID-19 related news and likely MDD. 

The psychological impacts of a public health emergency with a magnitude like COVID-19 are long lasting [58], and the psychosocial responses to the characteristic stressors it presents differ from country to country and person to person [59]. Respondents who were interested in receiving counselling were 2.56 times more likely to present with likely MDD compared with respondents who were not interested in receiving counselling. This finding is consistent with the results of a study conducted to evaluate the psychological impact of the COVID-19 epidemic on Guangdong college students [60]. This result could be due to the fact that people who perceive their psychological wellbeing as poor are more likely to seek counselling.

The Chi-squared analysis suggests that females had a higher prevalence of likely MDD (15.8%) compared to male respondents (8.8%). This finding is consistent with other studies during the COVID-19 pandemic, and during similar pandemics, which report that females have a higher prevalence of depression compared to males [8,50,61,62,63]. Women are more likely to be affected by mood and anxiety disorders during a pandemic, since depression and other mental health issues are more prevalent in women generally [64]. However, after controlling for other variables in the regression model, there was no statistically significant difference in the prevalence of likely MDD by gender in this study. Our finding is consistent with the cross-cultural WHO study on primary care, which revealed that depression with severe social disability was equally common among males as females and that gender ratios were dependent on the severity of depression [65]. Again, the DEPRES (Depression Research in European Society) study in its overall results stated that males and females who met either of the stem questions for depression manifested the same proportion of impairment (49%) [66].

In this study, age did not show an association with likely MDD when the age categories were examined with the Fisher’s exact test. This result is in contrast with studies that found a negative correlation between major depressive symptoms and age [16,67,68,69], in which age can present opportunities to acquire resilience due to exposure to stressors over time, leading to better coping skills [70]. On the contrary, in other studies, younger age [14] was associated with a higher prevalence of depressive symptoms in the COVID-19 pandemic [15]. The vulnerability of younger populations may be due to job-related uncertainty and the higher rates at which they are exposed to COVID-19 news on social media [32].

The Chi-squared analysis suggests that respondents who lived with family or friends had a higher prevalence of likely MDD compared to respondents with other housing characteristics. This result is consistent with an earlier study that found a higher prevalence of mental health problems such as depression due to conflicts between family members and friends living together during the COVID-19 pandemic [71]. This may be due to misunderstandings over whether or how family members and friends should protect themselves against the virus and the differences in opinions related to the conflicting information about the COVID-19 pandemic [71]. In another study, adequacy of living space, housing stability and housing satisfaction were significantly associated with a high prevalence of depression [72]. This reaffirms that housing is a key social determinant of mental health during the COVID-19 pandemic [73]. This notwithstanding, housing status did not independently predict depression in this study when other factors were controlled for in the regression model.

## 9. Strengths and Limitations

A major strength of the current study is the use of PHQ-9, a validated self-report scale with high reliability for the assessment of the primary outcome: likely MDD in the general population. Thus, it is expected that there would be a high correlation between the prevalence for likely MDD reported in this study and those that would have been reported using structured clinical interviews based on the Diagnostic and Statistical Manual for Mental Disorders 5, which is the gold standard for diagnosing MDD. Our study has a number of limitations. First, we used internet-based data collection methods and English language only survey questions which might have prevented individuals who wished to participate and had no internet access or who were not educated enough to read and write in English from taking part in the study. Second, distribution of survey links on WhatsApp groups means a large section of Ghanaians who were not members of these select social media groups or affiliated to members of the group were excluded from the survey. Third, after survey administration, the total number of completed responses was 756 which was less than our expected sample size. Using the online application (https://www.surveymonkey.com/mp/sample-size-calculator/ accessed on 1 May 2021), we determined that there was a 4% margin of error for our prevalence estimates for likely MDD which was much higher than the projected 3% margin of error based on our expected sample size. Fourth, the majority of respondents resided in the Ashanti region (64%), had a university or college education (85%) and were employed in a government Agency (57%), which is not representative of the demographic distribution of the Ghanaian population. Therefore, the results are not nationally representative and cannot be generalized. Fifth, all the variables were evaluated using self-reports and hence may suffer social desirability and recall biases. Finally, the cross-sectional nature of the survey does not allow for a direct causal relationship to be established between the variables included in the regression model and likely MDD. 

## 10. Policy Implications and Future Directions

There are important public health implications of the present study. The survey took place between the first and second waves of Ghana’s epidemic; therefore, this timing enables the researchers to evaluate the mental health impact that the lockdown and other public health preventive measures had on the general population. The government of Ghana could adopt for the population mental health interventions such as Text4Hope, launched in Alberta, Canada during the start of the COVID-19 pandemic, which effectively reduced depressive symptoms and suicidal ideation in subscribers [41,74,75,76]. Supportive text message programs are cost-effective, geographic location independent, are free to the end user and do not require expensive data plans, can reach thousands of people simultaneously and have been used to support residents of Alberta, Canada in managing stress, anxiety and depression [44,72,73,74,75,76,77,78,79,80]. A similar program could serve as a useful tool in Ghana and other low- and middle-income as well as high-income countries during the COVID-19 pandemic and also during natural disasters and other public health emergencies. 

## 11. Conclusions

To our knowledge, this is the first study to report on the prevalence and correlates of moderate/high depression symptoms among the general public in Ghana during the pandemic. Baseline prevalence for likely MDD for the general population are not currently available for comparison with the prevalence reported in this study. There is the urgent need for mental health officials, the ministry of health and the government of Ghana to have policies in place to alleviate the potential threat of COVID-19 on the mental health of the population. Implementing cost-effective, easily accessible and scalable population-level mental health programs such as supportive text message interventions could help provide residents of Ghana experiencing moderate/high depression symptoms with much-needed psychological support. 

## Figures and Tables

**Table 1:** Age distribution of demographic and social characteristics of respondents.

Variables	25 y or Less	26–40 y	41–60 y	More Than 60 y	Overall
**Gender**					
Male	25 (26.9%)	225 (52.9%)	72 (49.7%)	9 (42.9%)	331 (48.4%)
Female	68 (73.1%)	200 (47.1%)	73 (50.3%)	12 (57.1%)	353 (51.6%)
**Region**					
Ashanti	45 (54.2%)	280 (68.5%)	79 (57.2%)	14 (66.7%)	418 (64.2%)
Greater Accra	21 (25.3%)	95 (23.2%)	34 (24.6%)	6 (28.6%)	156 (24.0%)
Other	17 (20.5%)	34 (8.3%)	25 (18.1%)	1 (4.8%)	77 (11.8%)
**Education**					
Up to Junior High School	5 (6.0%)	13 (3.2%)	13 (9.4%)	5 (23.8%)	36 (5.5%)
Senior High School	25 (30.1%)	23 (5.6%)	7 (5.1%)	1 (4.8%)	56 (8.6%)
University/College/Post-Graduate	53 (63.9%)	372 (91.2%)	118 (85.5%)	15 (71.4%)	558 (85.8%)
**Relationship status**					
Single	59 (71.1%)	175 (42.8%)	9 (6.5%)	1 (4.8%)	244 (37.5%)
In relationship not married	20 (24.1%)	60 (14.7%)	2 (1.4%)	1 (4.8%)	83 (12.7%)
Married	4 (4.8%)	162 (39.6%)	120 (87.0%)	11 (52.4%)	297 (45.6%)
Divorce/Separated/Widowed	0 (0.0%)	12 (2.9%)	7 (5.1%)	8 (38.1%)	27 (4.1%)
**Employment status**					
Employed Govt Agency	20 (24.1%)	250 (61.1%)	94 (68.6%)	6 (28.6%)	370 (56.9%)
Private Agency	6 (7.2%)	80 (19.6%)	16 (11.7%)	1 (4.8%)	103 (15.8%)
Self Employed	3 (3.6%)	41 (10.0%)	20 (14.6%)	2 (9.5%)	66 (10.2%)
Unemployed	20 (24.1%)	28 (6.8%)	3 (2.2%)	3 (14.3%)	54 (8.3%)
Retired	0 (0.0%)	0 (0.0%)	3 (2.2%)	9 (42.9%)	12 (1.8%)
Student	34 (41%)	10 (2.4%)	1 (0.7%)	0 (0.0%)	45 (6.9%)
**Currently work in healthcare**					
Yes	21 (25.3%)	241 (58.9%)	74 (53.6%)	6 (28.6%)	342 (52.5%)
No	62 (74.7%)	168 (41.1%)	64 (46.4%)	15 (71.4%)	309 (47.5%)
**Health care profession**					
Physicians and physician assistants	3 (15.0%)	59 (25.3%)	34 (45.9%)	4 (66.7%)	100 (30.0%)
Nurses and Midwives	9 (45.0%)	107 (45.9%)	17 (23.0%)	0 (0.0%)	133 (39.9%)
Other Healthcare professionals	8 (40.0)	67 (28.8%)	23 (31.1%)	2 (33.3%)	100 (30.0%)
**Have had sufficient support from your employer during the pandemic**					
Yes, I have had absolute support	3 (4.1%)	71 (18.6%)	29 (22.3%)	2 (10.5%)	105 (17.4%)
Yes, I have had some support	7 (9.5%)	106 (27.8%)	51 (39.2%)	0 (0.0%)	164 (27.2%)
Yes, but only limited support	7 (9.5%)	75 (19.7%)	14 (10.8%)	1 (5.3%)	97 (16.1%)
No	21 (28.4%)	99 (26.0%)	21 (16.2%)	6 (31.6%)	147 (24.3%)
Not currently employed	36 (48.6%)	30 (7.9%)	15 (11.5%)	10 (52.6%)	91 (15.1%)
**Have had sufficient support from the Government of Ghana during the pandemic**					
Yes, I have had absolute support	13 (17.6%)	70 (18.4%)	25 (19.1%)	0 (0.0%)	108 (17.8%)
Yes, I have had some support	20 (27.0%)	128 (33.6%)	52 (39.7%)	6 (30.0%)	206 (34.0%)
Yes, but only limited support	19 (25.7%)	80 (21.0%)	20 (15.3%)	8 (40.0%)	127 (21.0))
No	22 (29.7%)	103 (27.0%)	34 (26.0%)	6 (30.0%)	165 (27.2%)
**Have had sufficient support from spiritual organizations and/or traditional healers/ mentors during the pandemic**					
Yes, I had absolute support	8 (10.8%)	68 (17.9%)	35 (26.7%)	6 (30.0%)	117 (19.3%)
Yes, I had some support	18 (24.3%)	82 (21.6%)	29 (22.1%)	5 (25.0%)	134 (22.1%)
Yes, but only limited support	8 (10.8%)	30 (7.9%)	14 (10.7%)	1 (5.0%)	53 (8.8%)
No	40 (54.1%)	200 (52.6%)	53 (40.5%)	8 (40.0%)	301 (49.8%)
**Have had sufficient support from family and friends during the pandemic**					
Yes, I had absolute support	32 (43.8%)	157 (41.1%)	60 (45.8%)	10 (50.0%)	259 (42.7%)
Yes, I had some support	19 (26.0%)	101 (26.4%)	30 (22.9%)	7 (35.0%)	157 (25.9%)
Yes, but only limited support	11 (15.1%)	29 (7.6%)	7 (5.3%)	0 (0.0%)	47 (7.8%)
No	11 (15.1%)	95 (24.9%)	34 (26.0%)	3 (15.0%)	143 (23.6%)
**Sought mental health counselling during the pandemic**					
Yes	2 (2.7%)	25 (6.6%)	6 (4.7%)	0 (0.0%)	33 (5.5%)
No	72 (97.3%)	355 (93.4%)	123 (95.3%)	20 (100.0%)	570 (94.5%)
**Have received mental health counselling during the pandemic**					
Yes	4 (5.5%)	52 (13.6%)	13 (10%)	0 (0.0%)	69 (11.4%)
No	69 (94.5%)	329 (86.4%)	117 (90%)	20 (100.0%)	535 (88.6%)

**Table 2:** Age distribution of social, clinical and COVID-19 related characteristics of respondents.

Variables	25 y or Less	26–40 y	41–60 y	More Than 60 y	Overall
**Would like to receive mental health counselling for psychological distress related to the pandemic**					
Yes	12 (16.2%)	54 (14.1%)	19 (14.5%)	1 (5.0%)	86 (14.1%)
Maybe	16 (21.6%)	125 (32.6%)	41 (31.3%)	3 (15.0%)	185 (30.4%)
No	44 (59.5%)	200 (52.2%)	71 (54.2%)	16 (80.0%)	331 (54.4%)
Currently receiving mental health counseling	2 (2.7%)	4 (1.0%)	0 (0.0%)	0 (0.0%)	6 (1.0%)
**Lost job due to the pandemic**					
Yes	2 (2.7%)	21 (5.5%)	5 (3.8%)	1 (5.0%)	29 (4.8%)
No	34 (45.9%)	334 (87.7%)	115 (88.5%)	12 (60.0%)	495 (81.8%)
Did not have a job before the COVID-19 pandemic	38 (51.4%)	26 (6.8%)	10 (7.7%)	7 (35.0%)	81 (13.4%)
**Frequency of watching television images of sick and dead people caused by COVID-19**					
Daily	29 (39.2%)	180 (47.0%)	73 (55.7%)	13 (65.0%)	295 (48.5%)
About every other day	17 (23.0%)	88 (23.0%)	26 (19.8%)	3 (15.0%)	134 (22.0%)
About once a week	8 (10.8%)	49 (12.8%)	16 (12.2%)	2 (10.0%)	75 (12.3%)
Less than once a week	8 (10.8%)	32 (8.4%)	7 (5.3%)	1 (5.0%)	48 (7.9%)
Did not watch images on any media of sick and dead people caused by COVID-19	12 (16.2%)	34 (8.9%)	9 (6.9%)	1 (5.0%)	56 (9.2%)
**Frequency of hearing radio stories of sick and dead people caused by COVID-19**					
Daily	36 (48.6%)	257 (67.1%)	101 (77.1%)	13 (65.0%)	407 (66.9%)
About every other day	22 (29.7%)	66 (17.2%)	16 (12.2%)	4 (20.0%)	108 (17.8%)
About once a week	11 (14.9%)	32 (8.4%)	10 (7.6%)	1 (5.0%)	54 (8.9%)
Less than once a week	3 (4.1%)	14 (3.7%)	3 (2.3%)	2 (10.0%)	22 (3.6%)
I did not watch or hear radio stories of sick and dead people caused by COVID-19	2 (2.7%)	14 (3.7%)	1 (0.8%)	0 (0.0%)	17 (2.8%)
**Frequency of reading newspaper stories, internet articles, or social media posts related to the pandemic**					
Daily	27 (37.0%)	224 (58.5%)	100 (76.3%)	16 (80.0%)	367 (60.5%)
About every other day	25 (34.2%)	90 (23.5%)	18 (13.7%)	3 (15.0%)	136 (22.4%)
About once a week	13 (17.8%)	35 (9.1%)	7 (5.3%)	1 (5.0%)	65 (9.2%)
Less than once a week	5 (6.8%)	29 (7.6%)	5 (3.8%)	0 (0.0%)	39 (6.4%)
Did not read news related to the pandemic	3 (4.1%)	5 (1.3%)	1 (0.8%)	0 (0.0%)	9 (1.5%)
**Have been fearful about contracting COVID-19**					
Yes	51 (68.9%)	263 (68.8%)	99 (75.6%)	13 (65.0%)	426 (70.2%)
No	23 (31.1%)	119 (31.2%)	32 (24.4%)	7 (35.0%)	181 (29.8%)
**Close friends or family members been sick from COVID-19**					
Yes	4 (5.4%)	122 (31.9%)	42 (32.1%)	2 (10.5%)	170 (28.1%)
No	70 (94.6%)	260 (68.1%)	89 (67.9%)	17 (89.5%)	436 (71.9%)
**Self-isolated or self-quarantined due to symptoms, recent travel, or contact with someone who may have COVID-19**					
Yes	4 (5.4%)	98 (25.7%)	28 (21.4%)	0 (0.0%)	130 (21.4%)
No	70 (94.6%)	284 (74.3%)	108 (78.6%)	20 (100.0%)	477 (78.6%)
**Worked in a designated holding/isolation centre or treatment centre as a health worker**					
Yes	5 (23.8%)	106 (45.3%)	29 (39.2%)	0 (0.0%)	140 (41.8%)
No	16 (76.2%)	128 (54.7%)	45 (60.8%)	6 (100.0%)	195 (58.2%)
**Likely Major Depressive Disorder**					
Yes	7 (11.9%)	44 (14.1%)	10 (9.2%)	0 (0.0%)	61 (12.3%)
No	52 (88.1%)	267 (85.9%)	99 (90.8%)	16 (100%)	434 (87.7%)

**Table 3:** Chi-squared and Fisher’s exact test, * test of association between the demographic, social and clinical antecedents and likely MDD.

Variables	Likely MDD Number (%)	*p*-Value
**Gender**		0.014
Male	21 (8.8%)	
Female	40 (15.7%)	
**Age (Years**)		0.139 *
≤25	7 (11.9%)	
26–40	44 (14.1%)	
41–60	10 (9.2%)	
>60	0 (0.0%)	
**Employment Status**		0.001 *
Government Agency	26 (9.1%)	
Private Agency	16 (19.8%)	
Self Employed	4 (7.7%)	
Unemployed	12 (29.3%)	
Retired	0 (0.0%)	
Student	3 (0.6%)	
**Relationship status**		0.056 *
Single	29 (15.6%)	
In a relationship but not married	11 (17.7%)	
Married	18 (8.0%)	
Divorced, Separated or Widowed	3 (14.3%)	
**Housing status**		0.032 *
Own home or mortgage	12 (11.1%)	
Renting accommodation	25 (9.5%)	
Live with family or friends	22 (20.6%)	
Housing not listed	2 (13.3%)	
**Region**		0.097 *
Ashanti	37 (11.6%)	
Greater Accra	20 (17.1%)	
Others	4 (6.9%)	
**Education**		0.783
Up to Junior High School	5 (16.1%)	
Senior High School	5 (13.2%)	
University/College/Post-Graduate	51 (12.0%)	
**Currently work in healthcare?**		0.046
Yes	24 (9.4%)	
No	37 (15.4%)	
**Health care profession**		0.814
Physicians/physician assistants	9 (11.1%)	
Nurses and midwives	8 (8.3%)	
Other healthcare professional	7 (9.1%)	
**Worked in a designated holding/isolation centre or treatment centre**		0.889
Yes	11 (9.7%)	
No	13 (9.2%)	
**Self-isolated or self-quarantined due to symptoms, recent travel, or contact with someone who may have COVID-19**		0.306
Yes	16 (15.2%)	
No	45 (11.5%)	
**Close friends or family members been sick from COVID-19**		0.120
Yes	22 (15.8%)	
No	38 (10.7%)	
**Have been fearful about contracting COVID-19**		0.049
Yes	49 (14.3%)	
No	12 (7.9%)	
**Frequency of reading newspaper stories, internet articles, or social media posts related to the pandemic?**		0.041 *
Daily	275 (90.2%)	
About every other day	96 (85.7%)	
About once a week	38 (84.4%)	
Less than once a week	20 (80.0%)	
I did not read news related to the pandemic	4 (57.1%)	
**Frequency of listening to radio stories of sick and dead people caused by COVID-19**		0.000 *
Daily	40 (12.1%)	
About every other day	9 (9.7%)	
About once a week	4 (9.5%)	
Less than once a week	0 (0.0%)	
Did not watch or hear radio stories of sick and dead people caused by COVID-19	8 (53.3%)	
**Frequency of watching television images of sick and dead people caused by COVID-19?**		0.005
Daily	19 (7.8%)	
About every other day	14 (12.5%)	
About once a week	9 (15.0%)	
Less than once a week	9 (24.3%)	
I did not watch images on any media of sick and dead people caused by COVID-19	10 (23.3%)	

* Fishers exact test.

**Table 4:** Chi-squared and Fisher’s exact test, * test of association between social and clinical antecedents and likely MDD.

Variables	Likely MDD Number (%)	*p*-Value
**Lost job due to the pandemic**		0.000
Yes	12 (46.2%)	
No	40 (9.9%)	
I did not have a job before the COVID-19 pandemic	9 (14.5%)	
**Have had sufficient support from family and friends during the pandemic**		0.930
Yes, I have had absolute support	25 (11.3%)	
Yes, I have had some support	16 (12.7%)	
Yes, but only limited support	5 (13.5%)	
No	15 (13.6%)	
**Have had sufficient support from spiritual organizations and/or traditional healers/ mentors during pandemic**		0.68
Yes, I have had absolute support	12 (12.1%)	
Yes, I have had some support	17 (15.0%)	
Yes, but only limited support	6 (13.3%)	
No	25 (10.5%)	
**Have had sufficient support from the Government of Ghana during the pandemic**		0.646
Yes, I have had absolute support	10 (10.4%)	
Yes, I have had some support	18 (10.8%)	
Yes, but only limited support	13 (13.1%)	
No	20 (15.0%)	
**Have had sufficient support from your employer during the pandemic**		0.109
Yes, I have had absolute support	9 (10.3%)	
Yes, I have had some support	13 (9.7%)	
Yes, but only limited support	6 (7.6%)	
No	19 (15.6%)	
Not currently employed	14 (19.7%)	
**Have sought mental health counselling during the pandemic**		0.151
Yes	6 (20.7%)	
No	54 (11.7%)	
**Have received mental health counselling during the pandemic**		0.810
Yes	8 (13.3%)	
No	53 (12.2%)	
**Would like to receive mental health counselling for psychological distress related to the pandemic**		0.035 *
Yes	15 (21.7%)	
Maybe	20 (13.7%)	
No	25 (9.1%)	
Currently receiving mental health counselling	1 (16.7%)	
**Received a mental health diagnosis from a health professional before the pandemic**		0.820 *
Yes	1 (10.0%)	
No	60 (12.4%)	
**Was on medication for a mental health concern before the pandemic**		0.621 *
Yes	0 (0.0%)	
No	1 (11.1%)	
**Drinking more alcohol than before the pandemic**		0.634 *
Yes, it is affecting my work, school, family, or social life	0 (0.0%)	
Yes, but it is not affecting my work, school family, or social life	0 (0.0%)	
No	32 (13.9%)	
No, did not drink alcohol even before the COVID-19 pandemic was declared	29 (11.3%)	
**Using cannabis (weed) more than before the pandemic**		0.793 *
Yes, and it is affecting my work, school, family, or social life	0 (0.0%)	
Yes, but it is not affecting my work, school, family, or social life	1 (25.0%)	
No	25 (13.3%)	
No, did not use cannabis even before the COVID-19 pandemic was declared	35 (11.6%)	
**Using drugs (excluding medication prescribed by a doctor) more than you used to before the pandemic**		0.750 *
Yes, and it is affecting my work, school, family, or social life	0 (0.0%)	
Yes, but it is not affecting my work, school, family, or social life	2 (22.2%)	
No	28 (13.0%)	
No, did not use drugs even before the COVID-19 pandemic was declared	31 (11.5%)	

* Fishers exact test.

**Table 5:** Logistic regression predicting likelihood for respondents to present with likely MDD.

Predictor	B	S.E.	Wald	Sig	EXP (B)	95% CI for EXP (B)	
						Lower	Upper
**Gender** **Female**	0.499	0.345	2.098	0.148	1.648	0.838	3.238
**Employment status**							
Govt Agency			7.014	0.220			
Private Agency	0.213	0.477	0.199	0.655	1.238	0.486	3.153
Self Employed	−1.893	0.954	3.937	0.047	0.151	0.023	0.977
Unemployed	0.657	0.689	0.909	0.340	1.928	0.500	7.435
Retired	−19.485	12,164.14	0.000	0.999	0.000	0.000	0.000
Student	−0.130	0.853	0.023	0.879	0.878	0.165	4.675
**Relationship status**							
Single			2.513	0.473			
In a relationship but not married	−0.219	0.497	0.194	0.660	0.804	0.304	2.127
Married	−0.692	0.453	2.331	0.127	0.500	0.206	1.217
Divorced, Separated or Widowed	0.076	0.819	0.009	0.926	1.079	0.217	5.377
**Work in Healthcare**							
No	0.425	0.457	0.865	0.352	1.529	0.625	3.743
**Housing status**							
Own home or mortgage			1.748	0.626			
Renting accommodation	−0.385	0.493	0.610	0.435	0.680	0.259	1.789
Live with family or friends	0.065	0.588	0.012	0.912	1.067	0.337	3.377
Housing not listed	0.282	0.915	0.095	0.758	1.326	0.221	7.964
**Friend/family sick from COVID**							
No	−0.203	0.397	0.262	0.609	0.816	0.374	1.778
**Fearful about getting COVID-19**							
No	−0.559	0.409	1.869	0.172	0.572	0.257	1.274
**Frequency of listening to COVID death news on radio**							
Daily			12.348	0.015			
Every other day	−0.988	0.503	3.857	0.050	0.372	0.139	0.998
Once a week	−2.175	0.850	6.547	0.011	0.114	0.021	0.601
Less than once a week	−19.780	10,208.31	0.000	0.998	0.000	0.000	0.000
Didn’t hear story on sick/dead	0.893	5.694	1.654	0.198	2.442	0.626	9.518
**Frequency of reading newspaper/social media posts**							
Daily			0.642	0.958			
Every other day	0.254	0.459	0.305	0.581	1.289	0.524	3.171
Once a week	0.419	0.604	0.480	0.488	1.520	0.465	4.969
Less than once a week	0.340	0.791	0.185	0.667	1.406	0.298	6.626
Didn’t read newspapers/posts	0.120	1.024	0.014	0.907	1.128	0.152	8.392
**Frequency of watching TV images of the sick/dead**							
Daily			7.502	0.112			
Every other day	0.669	0.492	1.853	0.173	1.952	0.745	5.116
Once a week	1.139	0.557	4.184	0.041	3.123	1.049	9.298
Less than once a week	1.341	0.627	4.573	0.032	3.824	1.118	13.072
Didn’t watch images on TV	1.221	0.627	3.797	0.051	3.392	0.993	11.588
**Lost job due to COVID-19**							
Yes			17.667	0.000			
No	−3.416	0.819	17.404	0.000	0.033	0.007	00.163
No job before COVID	−3.370	0.961	12.292	0.000	0.034	0.005	0.226
**Would like to receive mental health counselling**							
Yes			4.224	0.238			
Maybe	−0.633	0.491	1.667	0.197	0.531	0.203	1.388
No	−0.946	0.468	4.083	0.043	0.388	0.155	0.972
Currently receiving counseling	−0.192	1.291	0.022	0.881	0.825	0.066	10.354
**Constant**	1.745	1.138	2.349	0.125	5.726		

Abbreviations: SE, standard error; OR, odds ratio; and CI, confidence interval.

## Data Availability

Data for this study are available and can be released following reasonable request by writing to the corresponding author.

## References

[1] Lu H., Stratton C.W., Tang Y. (2020). Outbreak of pneumonia of unknown etiology in Wuhan, China: The mystery and the miracle. J. Med. Virol..

[2] Organization, W.H.O. (2020). COVID-19 Weekly Epidemiological Update.

[3] Ettman C.K., Abdalla S.M., Cohen G.H., Sampson L., Vivier P.M., Galea S. (2020). Prevalence of Depression Symptoms in US Adults Before and During the COVID-19 Pandemic. JAMA Netw. Open.

[4] Roberto K.J., Johnson A.F., Rauhaus B.M. (2020). Stigmatization and prejudice during the COVID-19 pandemic. Adm. Theory Prax..

[5] Huremović D. (2019). Social Distancing, Quarantine, and Isolation. Psychiatry of Pandemics.

[6] Guan W.J., Ni Z.Y., Hu Y., Liang W.H., Ou C.Q., He J.X., Liu L., Shan H., Lei C.L., Hui D.S.C. (2020). Clinical Characteristics of Coronavirus Disease 2019 in China. N. Engl. J. Med..

[7] Peeri N.C., Shrestha N., Rahman S., Zaki R., Tan Z., Bibi S., Baghbanzadeh M., Aghamohammadi N., Zhang W., Haque U. (2020). The SARS, MERS and novel coronavirus (COVID-19) epidemics, the newest and biggest global health threats: What lessons have we learned?. Int. J. Epidemiol..

[8] Özdin S., Özdin Ş.B. (2020). Levels and predictors of anxiety, depression and health anxiety during COVID-19 pandemic in Turkish society: The importance of gender. Int. J. Soc. Psychiatry.

[9] Verma S., Mishra A. (2020). Depression, anxiety, and stress and socio-demographic correlates among general Indian public during COVID-19. Int. J. Soc. Psychiatry.

[10] Mrklas K., Shalaby R., Hrabok M., Gusnowski A., Vuong W., Surood S., Urichuk L., Li D., Li X.-M., Greenshaw A.J. (2020). Prevalence of Perceived Stress, Anxiety, Depression, and Obsessive-Compulsive Symptoms in Health Care Workers and Other Workers in Alberta During the COVID-19 Pandemic: Cross-Sectional Survey. JMIR Ment. Health.

[11] Islam M.A., Barna S.D., Raihan H., Khan M.N.A., Hossain M.T. (2020). Depression and anxiety among university students during the COVID-19 pandemic in Bangladesh: A web-based cross-sectional survey. PLoS ONE.

[12] Muller R.A.E., Stensland R.S.Ø., van de Velde R.S. (2020). The mental health impact of the covid-19 pandemic on healthcare workers, and interventions to help them: A rapid systematic review. Psychiatry Res..

[13] Luo M., Guo L., Yu M., Jiang W., Wang H. (2020). The psychological and mental impact of coronavirus disease 2019 (COVID-19) on medical staff and general public—A systematic review and meta-analysis. Psychiatry Res..

[14] Ahmed M.Z., Ahmed O., Aibao Z., Hanbin S., Siyu L., Ahmad A. (2020). Epidemic of COVID-19 in China and associated psychological problems. Asian J. Psychiatry.

[15] Gao J., Zheng P., Jia Y., Chen H., Mao Y., Chen S., Wang Y., Fu H., Dai J. (2020). Mental health problems and social media exposure during COVID-19 outbreak. PLoS ONE.

[16] Nwachukwu I., Nkire N., Shalaby R., Hrabok M., Vuong W., Gusnowski A., Surood S., Urichuk L., Greenshaw A.J., Agyapong V.I. (2020). COVID-19 Pandemic: Age-Related Differences in Measures of Stress, Anxiety and Depression in Canada. Int. J. Environ. Res. Public Health.

[17] Agyapong V.I.O., Hrabok M., Vuong W., Gusnowski A., Shalaby R., Mrklas K., Li D., Urichuck L., Snaterse M., Surood S. (2020). Closing the Psychological Treatment Gap During the COVID-19 Pandemic With a Supportive Text Messaging Program: Protocol for Implementation and Evaluation. JMIR Res. Protoc..

[18] Fiorillo A., Gorwood P. (2020). The consequences of the COVID-19 pandemic on mental health and implications for clinical practice. Eur. Psychiatry.

[19] Chew N.W., Lee G.K., Tan B.Y., Jing M., Goh Y., Ngiam N.J., Yeo L.L., Ahmad A., Khan F.A., Shanmugam G.N. (2020). A multinational, multicentre study on the psychological outcomes and associated physical symptoms amongst healthcare workers during COVID-19 outbreak. Brain Behav. Immun..

[20] Liu S., Yang L., Zhang C., Xiang Y.-T., Liu Z., Hu S., Zhang B. (2020). Online mental health services in China during the COVID-19 outbreak. Lancet Psychiatry.

[21] Onchonga D., Ngetich E., Makunda W., Wainaina P., Wangeshi D., Viktoria P. (2021). Anxiety and depression due to 2019 SARS-CoV-2 among frontier healthcare workers in Kenya. Heliyon.

[22] Li Y., Scherer N., Felix L., Kuper H. (2021). Prevalence of depression, anxiety and post-traumatic stress disorder in health care workers during the COVID-19 pandemic: A systematic review and meta-analysis. PLoS ONE.

[23] Giannis D., Geropoulos G., Matenoglou E., Moris D. (2021). Impact of coronavirus disease 2019 on healthcare workers: Beyond the risk of exposure. Postgrad. Med. J..

[24] Hawryluck L., Gold W.L., Robinson S., Pogorski S., Galea S., Styra R. (2004). SARS Control and Psychological Effects of Quarantine, Toronto, Canada. Emerg. Infect. Dis..

[25] Bueno-Notivol J., Gracia-García P., Olaya B., Lasheras I., López-Antón R., Santabárbara J. (2021). Prevalence of depression during the COVID-19 outbreak: A meta-analysis of community-based studies. Int. J. Clin. Health Psychol..

[26] Nguyen H.C., Nguyen M.H., Do B.N., Tran C.Q., Nguyen T.T.P., Pham K.M., Pham L.V., Tran K.V., Duong T.T., Tran T.V. (2020). People with Suspected COVID-19 Symptoms Were More Likely Depressed and Had Lower Health-Related Quality of Life: The Potential Benefit of Health Literacy. J. Clin. Med..

[27] Semo B.-W., Frissa S.M. (2020). The Mental Health Impact of the COVID-19 Pandemic: Implications for Sub-Saharan Africa. Psychol. Res. Behav. Manag..

[28] Mazza C., Ricci E., Biondi S., Colasanti M., Ferracuti S., Napoli C., Roma P. (2020). A Nationwide Survey of Psychological Distress among Italian People during the COVID-19 Pandemic: Immediate Psychological Responses and Associated Factors. Int. J. Environ. Res. Public Health.

[29] Wang Y., Di Y., Ye J., Wei W. (2021). Study on the public psychological states and its related factors during the outbreak of coronavirus disease 2019 (COVID-19) in some regions of China. Psychol. Health Med..

[30] Lashitew A. (2020). Social distancing unlikely to hold up in Africa without a safety net for microentrepreneurs. Afr. Focus.

[31] Li W., Yang Y., Liu Z.-H., Zhao Y.-J., Zhang Q., Zhang L., Cheung T., Xiang Y.-T. (2020). Progression of Mental Health Services during the COVID-19 Outbreak in China. Int. J. Biol. Sci..

[32] Kazmi S.S.H., Hasan D.K., Talib S., Saxena S. (2020). COVID-19 and Lockdwon: A Study on the Impact on Mental Health. https://ssrn.com/abstract=3577515.

[33] Leonard B.E. (2001). The immune system, depression and the action of antidepressants. Prog. Neuro-Psychopharmacol. Biol. Psychiatry.

[34] Ma Y.-F., Li W., Deng H.-B., Wang L., Wang Y., Wang P.-H., Bo H.-X., Cao J., Wang Y., Zhu L.-Y. (2020). Prevalence of depression and its association with quality of life in clinically stable patients with COVID-19. J. Affect. Disord..

[35] Lee S.H., Shin H.-S., Park H.Y., Kim J.L., Lee J.J., Lee H., Won S.-D., Han W. (2019). Depression as a Mediator of Chronic Fatigue and Post-Traumatic Stress Symptoms in Middle East Respiratory Syndrome Survivors. Psychiatry Investig..

[36] Lee A.M., Wong J.G., McAlonan G.M., Cheung V., Cheung C., Sham P.C., Chu C.-M., Wong P.-C., Tsang K.W., Chua S.E. (2007). Stress and Psychological Distress among SARS Survivors 1 Year after the Outbreak. Can. J. Psychiatry.

[37] Wu K.K., Chan S.K., Ma T.M. (2005). Posttraumatic stress, anxiety, and depression in survivors of severe acute respiratory syndrome (SARS). J. Trauma Stress.

[38] Mak I.W.C., Chu C.M., Pan P.C., Yiu M.G.C., Chan V.L. (2009). Long-term psychiatric morbidities among SARS survivors. Gen. Hosp. Psychiatry.

[39] Tunvirachaisakul C., Gould R.L., Coulson M.C., Ward E., Reynolds G., Gathercole R.L., Grocott H., Supasitthumrong T., Tunvirachaisakul A., Kimona K. (2018). Predictors of treatment outcome in depression in later life: A systematic review and meta-analysis. J. Affect. Disord..

[40] Sharma A., Pillai D.R., Lu M., Doolan C., Leal J., Kim J., Hollis A. (2020). Impact of isolation precautions on quality of life: A meta-analysis. J. Hosp. Infect..

[41] Peng M., Mo B., Liu Y., Xu M., Song X., Liu L., Fang Y., Guo T., Ye J., Yu Z. (2020). Prevalence, risk factors and clinical correlates of depression in quarantined population during the COVID-19 outbreak. J. Affect. Disord..

[42] Shalaby R., Vuong W., Hrabok M., Gusnowski A., Mrklas K., Li D., Snaterse M., Surood S., Cao B., Li X.-M. (2021). Gender Differences in Satisfaction With a Text Messaging Program (Text4Hope) and Anticipated Receptivity to Technology-Based Health Support During the COVID-19 Pandemic: Cross-sectional Survey Study. JMIR mHealth uHealth.

[43] Agyapong V.I.O., Hrabok M., Vuong W., Shalaby R., Noble J.M., Gusnowski A., Mrklas K.J., Li D., Urichuk L., Snaterse M. (2020). Changes in Stress, Anxiety, and Depression Levels of Subscribers to a Daily Supportive Text Message Program (Text4Hope) During the COVID-19 Pandemic: Cross-Sectional Survey Study. JMIR Ment. Health.

[44] Nkire N., Mrklas K., Hrabok M., Gusnowski A., Vuong W., Surood S., Abba-Aji A., Urichuk L., Cao B., Greenshaw A.J. (2021). COVID-19 Pandemic: Demographic Predictors of Self-Isolation or Self-Quarantine and Impact of Isolation and Quarantine on Perceived Stress, Anxiety, and Depression. Front. Psychiatry.

[45] Abba-Aji A., Li D., Hrabok M., Shalaby R., Gusnowski A., Vuong W., Surood S., Nkire N., Li X.-M., Greenshaw A.J. (2020). COVID-19 Pandemic and Mental Health: Prevalence and Correlates of New-Onset Obsessive-Compulsive Symptoms in a Canadian Province. Int. J. Environ. Res. Public Health.

[46] Sapara A., Shalaby R., Osiogo F., Hrabok M., Gusnowski A., Vuong W., Surood S., Urichuk L., Greenshaw A.J., Agyapong V.I.O. (2021). COVID-19 pandemic: Demographic and clinical correlates of passive death wish and thoughts of self-harm among Canadians. J. Ment. Health.

[47] Kroenke K., Spitzer R.L., Williams J.B. (2001). The PHQ-9: Validity of a brief depression severity measure. J. Gen. Intern. Med..

[48] Sipsma H., Ofori-Atta A., Canavan M., Osei-Akoto I., Udry C., Bradley E.H. (2013). Poor mental health in Ghana: Who is at risk?. BMC Public Health.

[49] Solomou I., Constantinidou F. (2020). Prevalence and Predictors of Anxiety and Depression Symptoms during the COVID-19 Pandemic and Compliance with Precautionary Measures: Age and Sex Matter. Int. J. Environ. Res. Public Health.

[50] Annink A., Gorgievski-Duijvesteijn M., Dulk L.D. (2016). Financial hardship and well-being: A cross-national comparison among the European self-employed. Eur. J. Work. Organ. Psychol..

[51] Binder M. (2017). Entrepreneurial Success and Subjective Well-Being: Worries about the Business Explain One’s Well-Being Loss from Self-Employment. https://ssrn.com/abstract=3100865.

[52] Blanchflower D. (2004). Self-Employment: More May Not Be Better.

[53] Howe G.W., Hornberger A.P., Weihs K., Moreno F., Neiderhiser J.M. (2012). Higher-order structure in the trajectories of depression and anxiety following sudden involuntary unemployment. J. Abnorm. Psychol..

[54] Linn M.W., Sandifer R., Stein S. (1985). Effects of unemployment on mental and physical health. Am. J. Public Health.

[55] Mamun M.A., Sakib N., Gozal D., Bhuiyan A.I., Hossain S., Bodrud-Doza M., Al Mamun F., Hosen I., Safiq M.B., Abdullah A.H. (2021). The COVID-19 pandemic and serious psychological consequences in Bangladesh: A population-based nationwide study. J. Affect. Disord..

[56] Greene C.M., Murphy G. (2020). Individual differences in susceptibility to false memories for COVID-19 fake news. Cogn. Res. Princ. Implic..

[57] Chang J., Yuan Y., Wang D. (2020). [Mental health status and its influencing factors among college students during the epidemic of COVID-19]. Nan Fang Yi Ke Da Xue Xue Bao.

[58] Verghese A. (2004). What Is in a Word?.

[59] Liang S.-W., Chen R.-N., Liu L.-L., Li X.-G., Chen J.-B., Tang S.-Y., Zhao J.-B. (2020). The Psychological Impact of the COVID-19 Epidemic on Guangdong College Students: The Difference Between Seeking and Not Seeking Psychological Help. Front. Psychol..

[60] Alonso J., Angermeyer M.C., Bernert S., Bruffaerts R., Brugha T.S., Bryson H., de Girolamo G., de Graaf R., Demyttenaere K., The ESEMeD/MHEDEA 2000 Investigators (2004). Prevalence of mental disorders in Europe: Results from the European Study of the Epidemiology of Mental Disorders (ESEMeD) project. Acta Psychiatr. Scand..

[61] Liu N., Zhang F., Wei C., Jia Y., Shang Z., Sun L., Wu L., Sun Z., Zhou Y., Wang Y. (2020). Prevalence and predictors of PTSS during COVID-19 outbreak in China hardest-hit areas: Gender differences matter. Psychiatry Res..

[62] Alexander J.L., Dennerstein L., Kotz K., Richardson G. (2007). Women, anxiety and mood: A review of nomenclature, comorbidity and epidemiology. Expert Rev. Neurother..

[63] Maier W., Gänsicke M., Gater R., Rezaki M., Tiemens B., Urzúa R.F. (1999). Gender differences in the prevalence of depression: A survey in primary care. J. Affect. Disord..

[64] Angst J., Gamma A., Gastpar M., Mendlewicz J., Tylee A. (2002). Gender differences in depression. Eur. Arch. Psychiatry Clin. Neurosci..

[65] Christensen H., Jorm A., MacKinnon A.J., Korten A.E., Jacomb P.A., Henderson A.S., Rodgers B. (1999). Age differences in depression and anxiety symptoms: A structural equation modelling analysis of data from a general population sample. Psychol. Med..

[66] González-Sanguino C., Ausín B., Castellanos M.Á., Saiz J., López-Gómez A., Ugidos C., Muñoz M. (2020). Mental health consequences during the initial stage of the 2020 Coronavirus pandemic (COVID-19) in Spain. Brain Behav. Immun..

[67] Ozamiz-Etxebarria N., Dosil-Santamaria M., Picaza-Gorrochategui M., Idoiaga-Mondragon N. (2020). Stress, anxiety, and depression levels in the initial stage of the COVID-19 outbreak in a population sample in the northern Spain. Cad. Saude Publica.

[68] Birditt K.S., Fingerman K.L., Almeida D.M. (2005). Age Differences in Exposure and Reactions to Interpersonal Tensions: A Daily Diary Study. Psychol. Aging.

[69] Guo Y., Cheng C., Zeng Y., Li Y., Zhu M., Yang W., Xu H., Li X., Leng J., Monroe-Wise A. (2020). Mental Health Disorders and Associated Risk Factors in Quarantined Adults During the COVID-19 Outbreak in China: Cross-Sectional Study. J. Med. Internet Res..

[70] Suglia S.F., Duarte C.S., Sandel M.T. (2011). Housing Quality, Housing Instability, and Maternal Mental Health. J. Urban Health.

[71] Xie L., Zhou S., Zhang L. (2021). Associations between Objective and Subjective Housing Status with Individual Mental Health in Guangzhou, China. Int. J. Environ. Res. Public Health.

[72] Agyapong V., Shalaby R., Hrabok M., Vuong W., Noble J., Gusnowski A., Mrklas K., Li D., Snaterse M., Surood S. (2021). Mental Health Outreach via Supportive Text Messages during the COVID-19 Pandemic: Improved Mental Health and Reduced Suicidal Ideation after Six Weeks in Subscribers of Text4Hope Compared to a Control Population. Int. J. Environ. Res. Public Health.

[73] Agyapong V.I., Hrabok M., Shalaby R., Vuong W., Noble J.M., Gusnowski A., Mrklas K., Li D., Urichuck L., Snaterse M. (2021). Text4Hope: Receiving Daily Supportive Text Messages for 3 Months During the COVID-19 Pandemic Reduces Stress, Anxiety, and Depression. Disaster Med. Public Health Prep..

[74] Agyapong V.I.O. (2020). Coronavirus Disease 2019 Pandemic: Health System and Community Response to a Text Message (Text4Hope) Program Supporting Mental Health in Alberta. Disaster Med. Public Health Prep..

[75] Agyapong V.I., McLoughlin D.M., Farren C.K. (2013). Six-months outcomes of a randomised trial of supportive text messaging for depression and comorbid alcohol use disorder. J. Affect. Disord..

[76] Agyapong V.I.O., Milnes J., McLoughlin D.M., Farren C.K. (2013). Perception of patients with alcohol use disorder and comorbid depression about the usefulness of supportive text messages. Technol. Health Care.

[77] Agyapong V.I.O., Juhás M., Ohinmaa A., Omeje J., Mrklas K., Suen V.Y.M., Dursun S.M., Greenshaw A.J. (2017). Randomized controlled pilot trial of supportive text messages for patients with depression. BMC Psychiatry.

[78] Agyapong V.I.O., Mrklas K., Suen V.Y.M., Rose M.S., Jahn M., Gladue I., Kozak J., Leslie M., Dursun S., Ohinmaa A. (2015). Supportive Text Messages to Reduce Mood Symptoms and Problem Drinking in Patients With Primary Depression or Alcohol Use Disorder: Protocol for an Implementation Research Study. JMIR Res. Protoc..

[79] Agyapong V.I.O., Mrklas K., Juhás M., Omeje J., Ohinmaa A., Dursun S.M., Greenshaw A.J. (2016). Cross-sectional survey evaluating Text4Mood: Mobile health program to reduce psychological treatment gap in mental healthcare in Alberta through daily supportive text messages. BMC Psychiatry.

[80] Mao W., Agyapong V.I.O. (2021). The Role of Social Determinants in Mental Health and Resilience After Disasters: Implications for Public Health Policy and Practice. Front. Public Health.

